# Supplementary material for: How is physical healthcare experienced by staff, service users, and carers in adult community mental health services in a south London mental health trust? A service evaluation
Source: Front Health Serv. 2023 Jun 26;3:1125790. doi: 10.3389/frhs.2023.1125790 (PMC10335794; doi:10.3389/frhs.2023.1125790)
Supplement: Supplementary file 1 [file Datasheet1.docx]

Supplementary Material

**How is physical healthcare experienced by staff, service users, and carers in adult community mental health services in a South London Mental Health Trust? A Service Evaluation.**

Gracie Tredget*^1,5^†^,^ Julie Williams^2^†, Ray McGrath^1,5^, Euan Sadler^3^, Fiona Gaughran^1,4^, Karen Ang^1,5^, Natalia Stepan^5^, Sean Cross,^1,5^ John Tweed^1^, Lia Orlando^1^, Nick Sevdalis^2^.

*** Correspondence:** Gracie Tredget [gracie.tredget@slam.nhs.uk](mailto:gracie.tredget@slam.nhs.uk)

# 1. Supplementary Data

## 1.1. Clinical Staff Interview and Focus Group Topic Guide

**1. Physical health approach and practice**

1.1. Identify the standard journey for a patient accessing the community mental health team

*Prompts:*

- *How are the majority of patients referred to your team? (e.g., primary care, social care).*
- *What is the standard approach for patients that are referred: screening, assessment, allocation? Might physical health needs be identified at any of those stages? If so, what would typically happen?*
- *Would you accept a referral for a patient where physical health needs were identified as contributing to their mental health? If no, why not? If yes, how would you explore the comorbid concerns as a team?*
- *Does physical health feature in the decision when you allocate a patient to a Care Coordinator? How?*

1.2. Identify which roles are likely to support patients with physical health problems

*Prompts:*

- *Do you have any nominated champions of physical health that support you and the team? If yes, how are they appointed and what does their role involve? If no, why not?*
- *Do you work with teams in other organisations, such as primary care, social care, other hospitals, housing, around physical healthcare? How? Are there any challenges around this? What do you find helpful?*
- *Where do you go when you encounter a problem with patient’s physical health?*

1.3. Identify physical health priorities for the service

*Prompts:*

- *How much do you think the team prioritises physical health? Why?*
- *How are priorities set in the team? (E.g., are they discussed or mandated?) Are you asked to meet any targets/reporting around physical health? How are these communicated to you?*
- *How often does physical health come up in conversation with colleagues? Where does this come up? Prompt: team meetings, supervision*

1.4. Explore perceptions of physical healthcare practice within the team and across the Trust

*Prompts:*

- *Does your locality have its own strategy for physical health? What works well / not so well about your local approaches to physical health? Does this align to the Trusts?*
- *Do you know what the Trusts position on physical health is? Do you know where to find it?*
- *The new strategy states that physical health and mental health care will be treated equally. Do you agree with this approach? How confident are you that the Trust can realise this vision, and that these priorities will actually make a difference to patient care?*
- *Based on your experience do you face any particular challenges when putting into practice the physical health priorities of the Trust? What barriers do you foresee for physical healthcare in the future?*

**2. Use of physical health systems and tools**

2.1. Confirm the main databases, systems and tools used by the team (use checklist as prompt)

*Prompts:*

- *What is the main patient record system used by your team? How do you use this system when identifying/recording physical health needs?*
- *Does the system enable you to provide better physical healthcare? Is there anything you feel could improve experience for you/ your staff?*
- *Do your staff use any specific tools to help patients with physical healthcare needs?*
- *What experiences have you had / found your staff have had when using these tools?*
- *Do you use any additional systems/tools to identify, record, or report about physical health? If so, what do you use, and what is the staff experiences of using these?*

2.2. Communication of patient needs

*Prompts:*

- *Are there systems in place to be able to communicate easily with other professionals? If yes, which professionals do you work with (e.g., inside SLaM, primary care, acute care). Are there any barriers/facilitators to this?*
- *Is there anything else you are doing in your role or as a team/service to champion physical healthcare that you think other teams could learn from?*

**3. Physical health knowledge, skills, and training**

3.1 Identify the main problems experienced

*Prompts:*

- *What types of physical health problems do you work with your patients to manage?*
- *Do you feel that you have the adequate knowledge, skills, and training to deliver what is being asked of you by the patient/carer/team/Trust?*
- *What impact has this had on the way you approach or provide healthcare to your patients?*
- *Has it changed any interventions or how long you support a patient?*
- *Do you think staff feel able to engage confidently with patients about their physical health? Are there any barriers to this? What would help improve it?*

3.2. Explore barriers and facilitators to knowledge and skills

*Prompts:*

- *[Anything in addition to what we have already covered] What do you think you would need to be able to respond to physical health concerns? Prompt: skills, time*
- *What gaps are there? What else would you like staff to know? Why?*
- *What efforts have been made within your own team to address gaps or issues with knowledge and skills e.g., physical health leads, physical health forums etc. How helpful have these been, and why?*

3.3. Explore training experience

*Prompts:*

- *What training do you ask your staff / are you asked to do when joining the team? Prompt: part of induction, reviewed at appraisal, Mandatory Level 1 training*
- *Are there any additional tools (e.g., leaflets, apps etc.) that could help staff?*
- *Do you feel your team does anything specific to develop staff knowledge and skills around physical health? E.g., clinical / case reviews, business meetings, management rounds, supervision etc.*
- *[If not mentioned previously]. Is physical health competency or activity reviewed as part of your supervision? To what extent do you find this helps you to manage your development in this area?*

**4. Physical health attitudes, perceptions, and experiences of staff**

4.1. Identify the personal experiences and perspectives of the interviewee towards physical health

- *How important do you feel physical healthcare is in your role? Why is that? Any personal experiences you could draw upon?*
- *What has been your personal experience of supporting SMI patients with their physical health? What obstacles have you faced?*
- *What do you think the role of mental health staff should be in supporting people’s physical health? How successfully do you think staff achieve this in their roles now?*
- *Is there anything you hope to/would like to/think could be improved or changed about how physical healthcare is approached within your team / at SLaM? What do you think would be important when implementing these things?*

**5. Other (value added)**

*Prompts:*

- *What would you like to see from the Trust when looking at physical healthcare for the future?*
- *What more could the Trust do to engage staff around physical healthcare in the future?*
- *Examples of good practice*
- *Lessons that can be learned/shared (case study template)*

**6. Anything other business**

*Prompts:*

- *Areas for future research on physical health*
- *Anyone else that would be useful to speak to.*
- *Anything else that the interviewee feels has been missed and anything that they did not get a chance to discuss fully.*

# Service-User and Carer Focus Group Topic Guide

# Topic 1: Exploring your experience of physical healthcare when you have accessed an adult community mental health team.

- **Broad question:** What has your general experience of receiving physical healthcare been like when accessing SLaM Adult Community Mental Health Teams?
- **Narrow question:** Did you experience any problems when receiving physical healthcare? What worked well? What could help improve it?

**Topic 2:** Your perception of the knowledge and skills that mental health staff possess regarding physical health, and areas where you feel this could be improved.

- **Broad question:** To what extent do you think Adult Community Mental Health Team staff are adequately knowledgeable or trained on physical health issues?
- **Narrow question:** What knowledge, skills or training do you think staff could further benefit from to support adults with mental illness who have additional physical health problems?

**Topic 3:** The systems and tools that mental health staff use within the community mental health team to identify, monitor, record or communicate about a patient's physical healthcare needs (e.g., physical health questionnaires, physical health referrals).

- **Broad question:** Do you know why your CMHT holds data on your physical health needs? What difference do you think this makes to your care?
- **Narrow question:** How do you feel we engage with you about your physical health, using the information that you provide? How do you think the way we hold and share your data around physical health could be improved?

**Topic 4:** The team, service, and organisational approach towards physical healthcare and in particular, the culture this creates amongst staff, patients and carers, and the extent to which this aligns to the Trust’s strategic vision for physical healthcare.

- **Broad question:** As a service-user, to what extent do you understand the physical health priorities set out by the Trust? How much does this influence your engagement with SLaM for mental and physical health support?
- **Narrow question:** What do you think the future of physical healthcare at SLaM should look like or involve?
